# The Effects of Concurrent Training Combining Both Resistance Exercise and High-Intensity Interval Training or Moderate-Intensity Continuous Training on Metabolic Syndrome

**DOI:** 10.3389/fphys.2020.00572

**Published:** 2020-06-11

**Authors:** Marco Antônio R. Da Silva, Liliana C. Baptista, Rafael S. Neves, Elias De França, Helena Loureiro, Fabio Santos Lira, Erico C. Caperuto, Manuel T. Veríssimo, Raul A. Martins

**Affiliations:** ^1^Faculty of Sport Sciences and Physical Education, University of Coimbra, Coimbra, Portugal; ^2^Department of Physical Education, Universidade da Amazônia, Belém, Brazil; ^3^Department of Medicine, The University of Alabama at Birmingham, Birmingham, AL, United States; ^4^Human Movement Laboratory, São Judas Tadeu University, São Paulo, Brazil; ^5^School of Health Technologies, Polytechnic Institute of Coimbra, Coimbra, Portugal; ^6^Exercise and Immunometabolism Research Group, Department of Physical Education, Universidade Estadual Paulista (UNESP), Presidente Prudente, Brazil

**Keywords:** age groups, high-intensity interval training, resistance training, endurance training, metabolic syndrome

## Abstract

To date, there are several knowledge gaps on how to properly prescribe concurrent training to achieve the best dose-response, especially regarding the optimal intensity or volume of the aerobic component. Thus, the objective of this study is to analyze the effects of different aerobic exercise modes and intensities [i.e. aerobic high-intensity interval training (HIIT) versus moderate-intensity continuous aerobic training (MICT) combined with a resistance training (RT) program] on metabolic outcomes in participants with metabolic syndrome (MetS). Thirty-nine men and women (67.0 ± 6.7 years) volunteered to a 12-weeks exercise intervention (3 week^–1^, 50 min/session) and were randomly assigned to one of three groups: (a) RT plus MICT (RT+MICT) (2 males; 11 females); (b) RT plus HIIT (RT+HIIT) (4 males; 9 females); and (c) control group (CON) – without formal exercise (4 males; 9 females). Intensity was established between 60 and 70% of maximum heart rate (HRmax) in RT+MICT and ranged from 55–65% to 80–90% HRmax in the RT+HIIT group. Dependent outcomes included morphological, metabolic and hemodynamic variables. Both training groups improved waist circumference (RT+MICT: *P* = 0.019; RT+HIIT: *P* = 0.003), but not body weight, fat mass or fat-free mass (*P* ≥ 0.114). RT+HIIT group improved fasting glucose (*P* = 0.014), low density lipoprotein [LDL (*P* = 0.022)], insulin (*P* = 0.034) and homeostatic model assessment (*P* = 0.028). RT+MICT group reduced triglycerides (*P* = 0.053). Both exercise interventions did not change high sensitivity C-reactive protein, glycated hemoglobin, high density lipoprotein and total cholesterol, systolic, diastolic or mean arterial blood pressure (*P* ≥ 0.05). The CON group reduced the LDL (*P* = 0.031). This trial suggests that short-term exercise mode and intensity may differently impact the metabolic profile of individuals with MetS. Further, our data suggests that both concurrent trainings promote important cardiometabolic gains, particularly in the RT+HIIT. Nonetheless, due to the small-to-moderate effect size and the short-term intervention length, our data suggests that the intervention length also has an important modulating role in these benefits in older adults with MetS. Therefore, more research is needed to confirm our results using longer exercise interventions and larger groups.

## Introduction

Metabolic syndrome (MetS) is a combination of the most dangerous cardiovascular risk (CVR) factors including hyperglycemia, low density lipoprotein cholesterol (LDL-C), elevated triglycerides (TG), elevated systolic blood pressure (SBP) and increased waist circumference (WC) (Alberti et al., 2006). According to the International Diabetes Federation (IDF) MetS definition, almost 20–25% of the adult’s world population have MetS (Alberti et al., 2006) and those with MetS have an increased risk (∼ three times higher) of heart attack, stroke, type 2 diabetes (T2D), all-cause and cardiovascular death (Alberti et al., 2006; Sherling et al., 2017). Further, modifiable risk factors such as physical inactivity, diet and sedentary behavior have all been associated with the increase in MetS prevalence (Roberts and Barnard, 2005). Given the demographic shift (United Nations, 2009) and the prevalence of MetS in older adults, there is an urgent need of effective interventions to target MetS outcomes.

The increase in physical exercise has been recommended in prevention, primary treatment of cardiovascular disease (CVD) and MetS due to the cardioprotective benefits associated with the improvement of cardiorespiratory fitness (CRF) (Lakka and Laaksonen, 2007; Pedersen and Saltin, 2015; Ingle et al., 2017). On one hand, aerobic exercise promotes significant improvements in WC, fasting glucose, high density lipoprotein cholesterol (HDL-C), TG, diastolic blood pressure (DBP) and CRF in middle-aged and older adults (Wewege et al., 2018). On the other hand, resistance training (RT), an exercise regimen that increases muscle mass and strength, improves insulin sensitivity, enhances glucose oxidation (Bird and Hawley, 2017) and reduces the risk of premature death (Stamatakis et al., 2017). Notably, concurrent training, an integrative exercise modality that combines RT plus aerobic exercise may provide the benefits of both interventions (Stamatakis et al., 2017). When compared to control (no exercise) groups, concurrent training decreased WC, SBP and increased HDL-C and peak oxygen consumption (VO_2_peak) in patients with MetS (Ostman et al., 2017). However, a recent meta-analysis (Wewege et al., 2018) concluded that is required more studies with concurrent training to improve the quality of evidence on MetS risk factors.

Intensity is an important exercise prescription outcome, although to date, few studies using high-intensity interval aerobic training (HIIT) (Guadalupe-Grau et al., 2017; García-Pinillos et al., 2019) and moderate intensity continuous training [MICT (Ferrari et al., 2016)] have successfully been combined with resistance training (RT) program to improve neuromuscular and cardiorespiratory functions. However, to the best of our knowledge, there are no studies with RT+HIIT on MetS risk factors in older adults. Therefore, there is no empirical evidence to prescribe HIIT in detriment to MICT (or vice-versa) to improve MetS risk factors when associated to RT in older adults with MetS. Consequently, the aim of this study is to analyze the effects of different aerobic exercise modes and intensities (RT+HIIT versus RT+MICT) on metabolic outcomes in adults and older adults with MetS.

## Materials and Methods

### Study Design and Procedures

This clinical trial was conducted between May and September 2016 and the primary aim was to evaluate the effects of two modes and intensities of exercise, in MetS outcomes, in adults and older adults. This trial was developed at the Mealhada region, Portugal. Participants that agreed to participate in this study signed the informed consent form prior to study entry, consistent with the Declaration of Helsinki and later amendments (WMA, 2018). Furthermore, all methods and procedures were approved by the Ethic Committee of the Faculty of Sport Sciences and Physical Education of the University of Coimbra (FCDEF), Reference: CE/FCDEF-UC/00202016).

**FIGURE 1:**
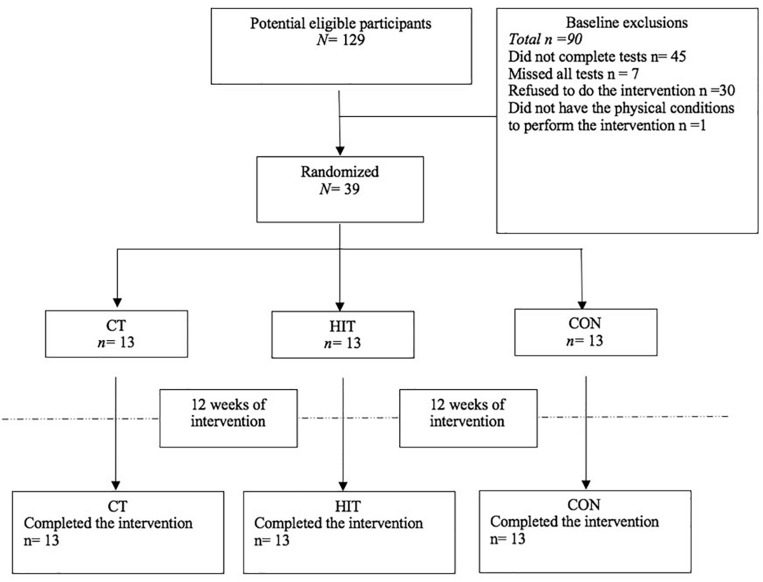
Cohort flux diagram.

Participants, were recruited from a public cardiology institution and were randomly assigned into one of 3 groups: (1) moderate-intensity continuous aerobic training (MICT) associate to resistance training (RT) (*n* = 13; 15% men); (2) High-intensity interval training (HIIT) associated to RT (*n* = 13; 31% men); and (3) Control group (CON), did not perform any formal exercise program (*n* = 13; 31% men) (Figure 1). Participants’ age ranged between 48 and 77 years old. All participants were sedentary (≤2 physical activity day per week ≤30 min per session), did not participate in structured training and presented at least 3 of the 5 MetS attributes.

The total exercise intervention length was of 12-weeks (3 sessions/week, 50 min/session). Intensity was established between 60 and 70% of maximal heart rate (HRmax) in RT+MICT group and varied between 55–65% and 80–90% of HRmax in the RT+HIIT group. The HRmax was estimated using Tanaka et al. (2001) equation (i.e. (208 – age) ^∗^ 0.7). Outcomes were evaluated at baseline and at the end of the intervention (12-weeks). Participants of the CON group did not engage in a formal exercise program during the intervention period and physical activity levels were self-reported by questionnaire at baseline and at the end of the intervention. Similarly, a self-reported dietary questionnaire was used to assess the food intake at baseline and after the intervention. All groups were instructed to maintain the same nutritional pattern throughout the trial and no changes in energy intake nor in macronutrients were reported.

After the recruitment period, participants completed the baseline testing including the measurement of the anthropometric and the hemodynamic profile, functional tests and CRF. All procedures were performed by specialized technical staff (nurses, physicians, health technicians and exercise physiologists), in appropriate facilities according to ACSM guidelines (Pescatello et al., 2014). Throughout the intervention, the same team of evaluators performed the measurements in the same order at baseline and after the 12-weeks of the intervention, to avoid evaluation errors and maintain the consistency of procedures. The technical staff was blinded for the post measurements once data was centralized in our laboratory and the staff responsible for the assessments was different from those responsible for running the exercise programs.

MetS was defined according to IDF criteria (Alberti et al., 2006). All participants had MetS according to IDF definition (IDF, 2006) and thus, had at least three or more of the following five attributes: (i) WC ≥ 94 cm for men or ≥80 cm for women; (ii) blood pressure above 130/85 mmHg; (iii) fasting blood glucose (FBG) ≥ 100 mg/dL; (iv) blood TG ≥ 150 mg/dL; and (v) HDL-C < 40 mg/dL for men and <50 mg/dL for women (Table 2). Participants were excluded according with the following criteria: (i) decompensated heart failure; (ii) angina pectoris; (iii) history of myocardial infarction or stroke with less than 1 year of evolution; and (iv) uncontrolled hypertension and self-reported renal failure.

### Intervention

The goal of this study is to determine the effect of different exercise modes and intensities of concurrent training (RT+MICT versus RT+HIIT) on MetS outcomes in adults and older adults. Exercise intervention occurred 3 times/week for approximately 50 min/session over 12 weeks in an enclosed gymnastic pavilion and the intervention combined RT followed by aerobic training (MICT or HIIT). The exercise sessions ended with a stretching session to promote cool-down.

The RT lasted approximately 20 min per session and included two sets of 8–15 repetitions, with a 1–2 min a rest interval (Pescatello et al., 2014). Intensity was measured with the Borg CR-10 scale (Borg, 1982), initially starting at 2 points and progressively increasing to five points weekly as described in Table 1. The RT exercises included: deadlift, barbell bent-over row, stiff-leg deadlift, bench press and crunches. It was used 1.20 m steel bars with plates, which were added as the exercise loads progressed. All the training sessions were supervised by an exercise physiologist who conducted the exercise sessions, motivated the participants and assured the correction and safety of movement execution.

**TABLE 1:** Periodization of strength training.

Weeks	Intensity (Borg CR-10)	Repetitions
1 and 2	2	15
3–5	3	12–15
6–10	4	10–12
11–12	5	8–10

**FIGURE 2:**
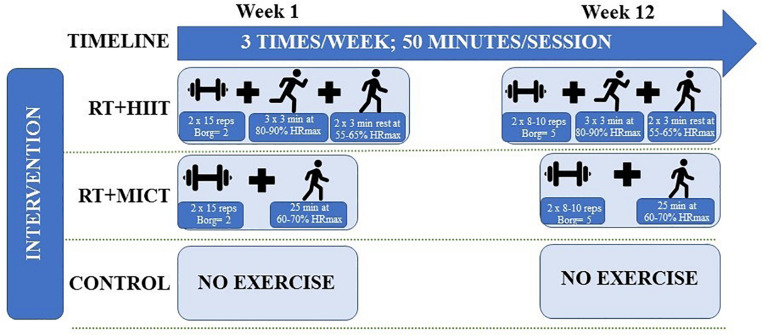
Illustrative scheme of exercise intervention across groups.

The aerobic exercise intervention (Figure 2) was designed to include different aerobic training protocols in each group. Participants in the RT+MICT group performed continuous aerobic training composed of 25 min of walking at moderate intensity (between 60 and 70% of the HRmax). Intensity was controlled by the rate of perceived exertion (RPE) using Borg CR-10 scale (Borg, 1982) and was objectively measured by a cardiac-telemetry device model ONRHYTHM 110, KALENJI^®^ (Villeneuve, France. The intensity of three participants under beta-blockers was controlled only through Borg CR-10 scale (Borg, 1982). The RPE started at level 3 and ended on 5th level at the end of the intervention. The RT+HIIT group performed aerobic exercise at a high- intensity interval composed of fast walking and running periods intercepted by 2 active recovery periods at moderate intensity, described as follow: participants run for 3 min, 3 times/session (those that were not able to run walked as fastest as they could) at 80–90% of HRmax. The high-intensity period was intercepted by an active recovery time in which participants walked moderately during 3 min at 55–65% of HRmax. Similarly, both the Borg CR-10 scale (Borg, 1982) and the cardiac-telemetry device were used to control the intensity in the RT+HIIT group. In the beginning of the intervention, the RPE started on 5th level and progressed until the 7th level in the end of the intervention. Similarly, the intensity set in the active recovery period was maintained between 2 and 3 points. In the last 5 min of each session, flexibility was used to promote cool-down and involved static movements of the large muscle groups, holding each position between 10 and 15 s, with 1–2 repetitions each exercise.

### Outcomes

#### Anthropometry

Body mass (BM) was measured in kilograms (kg) with a portable digital scale SECA^®^, model 770 (Hamburg, Germany), with a degree of accuracy of 100 g. Stature (S) was determined with the portable Harpenden stadiometer, model 98.603 (Crosswell, United Kindon), with a precision of 0.1 cm, and measured in meters (m). WC was determined with a plastic measuring tape HOLTAIN^®^ (Crosswell, United Kindon) with an accuracy of 0.1 cm (measured at the midpoint between the lower rib and the top of the iliac crest with relaxed abdomen). Relative and absolute fat mass (FM) and fat free mass (FFM) were determined using the dual energy radiological densitometry (DEXA) with a Lunar DXA System (version:13,6) manufactured by GE Healthcare (United States), and with the results expressed in kg. Body mass index (BMI) was calculated dividing the BM in kilograms by S in square meters (kg/m^2^).

#### Hemodynamics

Blood pressure was measured using a digital sphygmomanometer tavolo with a 4” LCD display (Moretti S.p.A.), model DM460 (Arezzo, Italy) and the results were expressed in millimeters of mercury (mmHg). Participants rested 5 min in the sitting position and afterward, two measurements were performed with 1-min interval between both evaluations. SBP and DBP were calculated using the mean values of the 2 evaluations. Mean arterial pressure (MAP) was calculated using the formula MAP = SBP + (DBP × 2)/3.

#### Blood Analysis

The blood samples were collected by trained nurses in the morning after 12 h of fasting and 48 h after the last training session. The variables were determined immediately after blood collection (without freezing) by standard methods performed in an accredited laboratory of clinical analysis (Laboratório UÁLIA – Análises Clínicas). Blood serum (centrifugate at 3500 RPM) were used for LDL-C, HDL-C, total cholesterol (TC), TG, FBG and high-sensitivity C-reactive protein (hs-CRP) quantification using Beckman Coulter AU 400 (Brea, United States) commercial kits. Further, blood serum samples were used to quantify insulin and C-peptide (CP) using the Roche Cobas (Mannheim, Germany) commercial kits. Whole blood was used for glycated hemoglobin (HbA1c) using Menarini–Arkray HA 8140 (Kyoto, Japan) commercial kit. Error analysis was less than 1 standard deviation for all variables (LDL-C, HDL-C, TC, TG, hs-CRP, CP, FBG and HbA1C). The evaluation of the homeostasis model of insulin resistance (HOMA-IR) was also used to determine insulin resistance and was calculated by a free online calculator (HOMA Calculator, Version 2.2.3, University of Oxford, Oxford, United Kingdom).

### Sample Size Calculation

Originally, our study was designed to assess changes in CRF in RT+HIIT versus MICT+RT. We used an anticipated mean difference between groups from a prior systematic review with meta-analysis (Weston et al., 2014) with a similar population (i.e. in hypertensive older adults) and with an exercise intervention length of 4 to 16 weeks to calculate our sample size. Therefore, to identify a mean difference of 3.3 ± 3.03 mL/kg^–1^/min^–1^ between MICT and HIIT groups using a two-sided significance level of 0.05 and assuming 80% power (β = 0.80), we would require 17 participants in each group. Further, based on data from the study of Robinson et al. (2017) that identified an increase of +4.4 [1.8–6.9] mL/kg^–1^/min^–1^ in VO_2_peak in an older adult sample after 12 weeks of RT + HIIT, and using the same statistical criteria (alpha 0.05 and beta 0.80 with paired t-test) to identify a mean difference between groups, a total of 10 participants are required. We recruited 129 volunteers (but only 39 participated in the intervention, see Figure 1), therefore that was not enough to account for potential attrition rates for between groups difference (*N* = 17; with α = 0.05 and β = 0.80), as only 13 participants remained in each group this was sufficient for pre- to post intervention (*N* = 10; with α = 0.05 and β = 0.80).

### Statistical Analysis

Data are presented as mean followed by standard deviation (SD) or when specified by standard error. Pre- to pos-intervention differences on clinical outcomes were measured in the three groups. Participants baseline characteristics were described using differences between means and SD for the following variables: age, BM, WC, BMI, VO_2_peak, SBP, DBP, TC, HDL-C, LDL-C, TG, FBG, HbA1C.

The assumption of normality was checked using visual histogram analysis and confirming if the asymmetry and kurtosis were within the acceptable range (i.e. between +3 and −3) (Lomax and Schumacker, 2004; Hair et al., 2006). When variables assumed levels of significance higher than 0.05 it was also used the Shapiro-Wilks hypothesis test, to confirm normal distribution.

Differences between groups at baseline were assessed through analysis of variance (ANOVA) using pairwise comparisons and through multivariate analysis of variance (MANOVA). When a significance level of *P* < 0.05 was identified, the Bonferroni *post hoc* test was used to identify differences between groups and to correct for multiple comparisons. In addition, the analysis of covariance (ANCOVA) was also performed to adjust for the effect of sex and age between groups.

A two-way ANOVA for repeated measures was used to test the differences between groups and to compare the effects of pre- to post-intervention. Similarly, when *P* < 0.05 was identified, the Bonferroni *post hoc* test was used to identify differences between groups and to control for multiple comparisons. The magnitude of the effect was calculated using Cohen’s *d* effect size (Cohen, 1988) to compare within groups changes (pre to post-intervention) and eta squared (η2) for ANOVA repeated measures. The estimation of the Cohen’s *d* effect size was categorized according to the following criteria: small (<0.20), moderate (0.20–0.79), and large (>0.80) (Cohen, 1988). Eta squared with 0.06, 0.06–0.14, and >0.14 was categorized as small, medium, and large effect, respectively.

All statistical analyzes were performed using the software Statistical Package for the Social Sciences for Windows (IBM-SPSS, Inc., Chicago, IL, United States, EUA), version 24.0. The significance level was set at 95%.

## Results

### Sample Characteristics

One hundred and twenty-nine potential eligible participants were recruited. After applying the inclusion and exclusion screening criteria, 39 participants (67.0 ± 6.7 years) were randomly assigned to one of three groups: a) RT+MICT (*n* = 13; 15% Men); b) RT+HIIT+ RT (*n* = 13; 31% Men); and c) CON (*n* = 13; 31% Men). All participants completed the 12-weeks of the intervention and were included in the analysis (Figure 1).

#### Within and Between Group Comparison

The demographic characteristics and differences between groups at baseline in CRF, anthropometric and hemodynamic variables are described in Table 2. At baseline, the demographic and clinical characteristics between groups were similar, except in age and gender. Participants in the RT+HIIT group were younger than the RT+MICT group (*P* ≤ 0.05).

**TABLE 2:** Demographic and clinical characteristics of participant’s at baseline and comparison between groups calculated from a MANOVA controlling for the effect of sex and age.

	RT+MICT (*n* = 13)	RT+HIIT (*n* = 13)	CON (*n* = 13)	*P*
Women, *n* (%)	11(85%)	9(69%)	9(69%)	0.584^#^
Age, years	71.1(4.8)	63.3 (7.2)*	67.4(3.9)	**0.001**
Body mass, kg	70.5(14.6)	77.9(17.4)	76.1(9.3)	0.596
BMI, kg/m^2^	29.3(5.5)	31.1(5.5)	29.5(3.2)	0.633
WC, cm	98.2(11.5)	102.5(14.7)	99.8(9.0)	0.788
VO_2_peak, mL/kg^–1^/min^–1^	19.7(3.9)	21.4(4.0)	20.5(3.3)	0.577
Fat mass, %	38.6(4.6)	39.5(7.3)	38.2(6.2)	0.554
Fat mass, Kg	27.3(7.2)	31.0(11.2)	28.6(4.7)	0.529
Fat free mass, Kg	39.9(8.9)	43.7(8.3)	44.3(8.3)	0.601
SBP, mmHg	135.2(14.1)	132.4(19.7)	129.7(12.1)	0.574
DBP, mmHg	75.2(10.1)	77.7(9.7)	70.8(7.8)	0.114
MAP, mmHg	95.2(10.5)	95.9(12.4)	90.5(8.7)	0.276
Glucose, mg/dl	89.8(11.9)	97.2(29.5)	95.4(12.2)	0.548
HbA1c, %	5.4(0.4)	5.8(0.8)	5.4(0.3)	0.096
Total colesterol, mg/dl	185.2(26.9)	190.4(34.0)	191.0(23.7)	0.669
HDL, mg/dl	53.5(19.7)	56.7(12.2)	54.9(8.8)	0.709
LDL, mg/dl	109.23(24.7)	116.9(27.0)	120.9(18.9)	0.334
TG, mg/dl	113.2(35.9)	119.4(44.2)	119.2(62.8)	0.980
hsCRP, mg/dl	0.32(0.4)	0.46(0.4)	0.15(0.1)	0.144
Insulin, mUI/l	12.8(8.7)	16.5(13.1)	8.8(3.5)	0.126
Peptide C, ng/ml	2.0(0.9)	1.9(0.9)	2.0(0.5)	0.751
HOMA-IR	1.65(1.1)	2.12(1.6)	1.10(0.4)	0.098
**Metabolic syndrome – IDF criteria**				
Central obesity (37)	11	13	13	0.312^#^
Raised blood pressure (24)	8	10	6	0.377^#^
Dyslipidemia (20)	12	6	2	**0.020**^#^
Raised fasting plasma glucose (FPG) (8)	2	3	3	1.00£
Reduced HDL (12)	6	2	4	0.236^#^
Raised TG (7)	2	2	3	1.00£
BMI obesity (18)	7	6	5	0.446^#^
Diabetes Melitus T2 (5)	1	2	2	1.00£

*Data are expressed as mean (± SD) or percentage as appropriate (%). ^∗^*P* ≤ 0.05, when compared to other two groups; ^#^chi-square test; £, Monte Carlo test. BM (Body mass); BMI (Body mass index); DBP (Diastolic blood pressure); FM (Fat mass); FPG (Fasting plasma glucose); HbA1c (Glycosylated Hemoglobin); HDL (High Density Lipoprotein Cholesterol); HOMA-IR (homeostasis model of insulin resistance; hsCRP (high-sensitivity C-reactive protein); LDL (Low Density Lipoprotein Cholesterol); MAP (Mean arterial pressure); SBP (Systolic blood pressure); TC (Total cholesterol); TG (Triglycerides).*

From pre- to post-intervention, there were a large and significant main effect of time in the metabolic outcomes for WC (*F* = 23.689, *P* = 0.000, η2 = 0.397), HDL-C (*F* = 4.766, *P* = 0.034, η2 = 0.117), TG (*F* = 4.590, *P* = 0.053, η2 = 0.277), insulin (*F* = 5.728, *P* = 0.034, η2 = 0.323), HOMA-IR (*F* = 7.120, *P* = 0.020, η2 = 0.372) and a trend for FBG (*F* = 3.289, *P* = 0.078, η2 = 0.084), CP (*F* = 3.651, *P* = 0.080, η2 = 0.233). There was a large group interaction in LDL-C (*F* = 3.453 *P* = 0.042, η2 = 0.161). There was no effect of time (*P* > 0.05) or group interaction (*P* > 0.05) for BM, BMI, hs-CRP, HbA1c, TC, SBP, DBP, FM (% and kg) and FFM. After the intervention, participants in the RT+MICT and RT+HIIT group moderately decreased WC (*P* = 0.019 and *P* = 0.003, respectively). Participants in the RT+HIIT group moderately decreased LDL-C (*P* = 0.022) and had a small decrease in fasting glucose (*P* = 0.014), insulin (*P* = 0.034) and HOMA-IR (*P* = 0.028). Furthermore, in the RT+MICT group, there was a trend to reduce HDL-C (*P* = 0.072) and CP (*P* = 0.080) and a significant decrease in TG (*P* = 0.053). Participants in the CON group had a significant reduction only in LDL-C (*P* = 0.031). There was no difference (*P* ≥ 0.09) between groups after the period of intervention. Statistical data are presented in Table 3 and as scatterplots in Supplementary Datasheet 1.

**TABLE 3:** Differences between pre- and post-intervention and between groups of the morphological and hemodynamic outcomes calculated with two-way analyses of variance (ANOVA) for repeated measures.

	RT+MICT (*n* = 13)	*P* within group	ES	RT+HIIT (*n* = 13)	*P* within group	ES	CON (*n* = 13)	*P* within group	ES	*P* between groups	Observed Power within group	Observed Power between groups
BM	−0.724(0.5)	0.216	–0.05	−1.16(0.6)	0.085	–0.08	0.340(0.6)	0.558	0.01	0.418	0.249	0.349
BMI, kg/m^2^	−0.16(0.5)	0.749	–0.03	−0.44(0.2)	0.145	–0.08	0.05(0.1)	0.588	0.02	0.354	0.150	0.130
WC, cm	−3.61(1.3)	**0.019**	–0.31	−4.80(1.2)	**0.003**	–0.32	−2.61(1.5)	0.111	–0.35	0.716	0.994	0.147
FM, %	−0.03(0.3)	0.924	0.01	−0.46(0.6)	0.480	–0.07	0.26(0.3)	0.413	0.04	0.922	0.055	0.157
FM, kg	−0.48(0.4)	0.335	–0.07	−0.51(0.6)	0.436	–0.05	0.24(0.2)	0.279	0.05	0.531	0.139	0.173
FFM, kg	0.66(1.0)	0.519	0.08	−0.93(0.5)	0.114	–0.11	−0.21(0.2)	0.431	–0.03	0.445	0.445	0.236
SBP, mmHg	−3.96(3.7)	0.312	–0.34	2.65(6.2)	0.679	0.15	2.46(3.7)	0.520	0.20	0.846	0.052	0.148
DBP, mmHg	−0.38(1.9)	0.849	–0.04	−1.42(2.5)	0.590	–0.15	3.2(2.7)	0.255	0.43	0.576	0.063	0.212
MAP, mmHg	−1.57(2.1)	0.486	–0.18	−0.06(3.6)	0.986	–0.01	3.00(2.7)	0.294	0.36	0.790	0.058	0.148
Glucose, mg/dl	−1.53(2.2)	0.512	–0.12	−5.00(1.7)	**0.014**	–0.17	−0.23(2.4)	0.925	0.09	0.704	0.287	0.373
HbA1c, %	−0.06(0.6)	0.312	0.14	−0.07(0.5)	0.201	–0.09	0.02(0.3)	0.553	–0.16	0.091	0.092	0.371
TC, mg/dl	−2.53.(7.4)	0.738	–0.10	−7.69(9.2)	0.422	–0.25	−1.23(7.3)	0.870	–0.05	0.393	0.126	0.076
HDL, mg/dl	−4.15(2.1)	0.072	–0.23	−2.53(1.5)	0.116	–0.20	−0.53(1.5)	0.762	–0.06	0.730	0.642	0.223
LDL, mg/dl	4.92(6.4)	0.393	0.20	−14.00(5.7)	**0.022**	–0.55	−12.15(4.9)	**0.031**	–0.29	0.847	0.565	0.610
TG, mg/dl	−17.61(8.2)	**0.053**	0.55	9.00(10.5)	0.566	0.18	28.23(15.5)	0.077	0.24	0.534	0.565	0.610
hsCRP, mg/dl	−0.14(0.1)	0.155	–0.46	−0.03(0.1)	0.814	–0.06	0.01(0.1)	0.560	0.08	0.328	0.109	0.420
Insulin, mUI/l	−0.34(1.6)	0.858	–0.04	−2.12(0.8)	**0.034**	–0.16	0.59(1.5)	0.754	0.12	0.239	0.055	0.319
Peptide C, ng/ml	−0.25(0.1)	0.080	–0.28	−0.16(0.1)	0.193	–0.18	0.12(0.2)	0.638	0.14	0.164	0.050	0.799
HOMA-IR	−0.05(0.2)	0.824	–0.04	−0.28(0.1)	**0.028**	–0.18	0.07(0.2)	0.729	0.13	0.288	0.077	0.310

*Data are expressed as differences of means followed by standard error; ES (Effect size); BM (Body mass); BMI (Body mass index); DBP (Diastolic blood pressure); FFM (Free fat mass)FM (Fat mass); HbA1c (Glycosylated Hemoglobin); HDL (High Density Lipoprotein Cholesterol); HOMA-IR (homeostasis model of insulin resistance); hsCRP (high-sensitivity C-reactive protein); LDL (Low Density Lipoprotein Cholesterol); MAP (Mean arterial pressure); SBP (Systolic blood pressure); TC (Total cholesterol); TG (Triglycerides).*

## Discussion

The health benefits of exercise training are indisputable in the prevention and management of many age-related diseases (Pedersen and Saltin, 2015). Exercise was also associated to positive effects on MetS, a multifactorial syndrome that encompasses at least three of the five most prevalent CVD risk factors including hypertension, diabetes, hypercholesterolemia, visceral adiposity and low HDL-C (Lakka and Laaksonen, 2007; Pedersen and Saltin, 2015; Ingle et al., 2017). However, to date there are several knowledge gaps and inconsistencies in the literature regarding the impact of exercise mode and intensity effects on MetS outcomes (Wewege et al., 2018). Thus, we developed a 12-week randomized trial to evaluate the effects of two different exercise modes and intensities on metabolic outcomes. After the intervention, our results suggest that when compared to pre-intervention, 12-weeks of concurrent training positively impact the metabolic profile of individuals with MetS. Further, our data also suggests that the mode and intensity of exercise training may differently impact the metabolic outcomes in individuals with MetS, i.e. despite the small-to moderate-effect, RT+HIIT may potentially improve WC, LDL-C, FBG, insulin and HOMA-IR profile at greater extension than RT+MICT in adults and older adults with MetS. Multiple evidence confirms that older adults have good physiological, molecular and mechanical responses to HIIT as younger individuals (Jabbour et al., 2017; Robinson et al., 2017; Yasar et al., 2019). Therefore, despite youngers, the RT+HIIT revealed a small to moderate effect which might have been limited by the short-term intervention rather than by the age range.

Our short-term (12 week) RT+MICT program promoted several metabolic changes in MetS markers that are consistent with previous studies (Robinson et al., 2017; Agner et al., 2018; Banitalebi et al., 2018; Cadore et al., 2018). In contrast, Banitalebi et al. (2018) compared the effect of 12-weeks (3 times/week) of RT (16–18 RM to 8–10 RM) with cycling continuous aerobic exercise (16 to 30 min at 60–90% HRmax) in older women and did not found any significant difference in several endocrine outcomes including insulin like growth factor-1, cortisol and insulin profile. Similarly, Agner et al. (2018) reported no significant change in blood cholesterol and glucose outcomes after 12 weeks (2 sessions/week) of concurrent training (50 min of RT at 40–70% 1RM and 40 min of walking exercises at 70–85% HRmax). Further, in a recent study with high volume, Robinson et al. (2017) did not found any improvement in insulin sensitivity after 12 weeks of RT+ MICT (MICT, 30 min at 70% VO_2_peak, 5 day/week; RT, 2–4 sets per exercise, 4 times/week) (Robinson et al., 2017). Similarly, in a recent meta-analysis (Ostman et al., 2017) with 4 studies using concurrent training with a 3–13 months duration (MICT followed by RT), Ostman et al. (2017) showed that RT+MICT decreased SBP and WC, increased HDL-C, and unchanged FPG, TG and LDL-C. Collectively, these results suggest that the intervention length is an important mediator in RT+MICT, indicating that greater gains require long-term exercise training. Notably, the results shown by Balducci et al. (2010) seem to support this rationale once the long-term (12-months) concurrent training decreased HbA1c, hs-CRP, HOMA-IR and increased HDL-C. However, there were no changes in LDL-C. In contrast, our short-term RT+HIIT (but not RT+MICT) showed small-to moderate effects on abdominal adiposity, FBG, insulin and HOMA-IR in older adults with MetS. Therefore, more studies are needed to clarify effects of long-term RT+HIIT in older adults with MetS.

Remarkable evidence from the “omics” exercise area (i.e. epigenetics, transcriptome and metabolomics) (Robinson et al., 2017) suggest that the mode and intensity of concurrent training may up-regulate different metabolic responses in older adults with MetS and may explain the positive physiological and molecular mechanisms in response to exercise intervention in our trial. For instance, aerobic exercise enhances mitochondrial oxidative enzymes’, which in turn are associated with improvements in insulin sensitivity with age (Lanza et al., 2008). On the other hand, RT reverses age-related declines in myosin heavy-chain gene transcripts and increases muscle protein synthesis rates, improves skeletal muscle mass quality and function leading to enhanced glucose oxidation and improved insulin sensitivity (Bird and Hawley, 2017). Therefore, concurrent training, an exercise modality that combines both modes of exercise, can potentially upregulate many physiological mechanisms of both interventions.

Despite multiple cardiometabolic benefits of exercise training, evidence suggests that when compared to HIIT, lower intensities may limit mitochondrial activity in healthy populations (Lanza et al., 2008; Robinson et al., 2017), Thus, exercise intensity plays an important role mediating insulin sensitivity (Robinson et al., 2017; Liu et al., 2019). Several studies using lower intensities confirm the inexistence of effects on insulin sensitivity with concurrent training (Robinson et al., 2017). Notably, our results seem to support and confirm this rationale once both exercise groups (RT+MICT; 60–70% HRmax and RT+HIIT; 80–90% HRmax) exhibited different insulin responses. There were no significant changes on RT+MICT in FBG, insulin and HOMA-IR while in the RT+HIIT there were several improvements on metabolic outcomes important to the regulation of insulin metabolism including WC, FBG, insulin and HOMA-IR. Notably, the RT+MICT group only decreased WC and TG. Physiologically, lower exercise intensities limit mitochondrial oxidative enzymatic capacity which in turn, lead to the decrease in glucose uptake by skeletal muscle leading to the increase in plasmatic glucose. These deleterious effects decrease insulin sensitivity and increase glucose deposition in adipocytes (Bird and Hawley, 2017; Robinson et al., 2017). In contrast, high intensity may revert this process leading to the increase in insulin sensitivity (i.e. HOMA-IR) and to a decrease in circulating indices of glucose and, in fat deposition. Clinically, these results have a significant effect on CVD regression, particularly by decreasing several metabolic markers of MetS and of T2D, a prevalent morbidity associated with MetS (Alberti et al., 2006).

Interestingly, our exercise protocols did not promote any change in TC, HDL-C and TG across groups. The RT+HIIT and CON group only decreased LDL-C (12 and 10%, respectively). Potential explanations may include the nutritional pattern and use of pharmacological treatment (Supplementary Table S1). Despite instructions to maintain a similar nutritional pattern throughout the study, and the self-reported unchanged, participants tend to be more careless with daily dietary quality and calorie ingestion thinking that exercise may compensate for these differences. In addition, the use of several lipid lowering drugs may also explain, at least in part, the results in the CON group once the improvements in LDL-C were not visible in other lipid components (i.e. HDL-C and TG). Further, the intervention length may have also contributed as an important modulator factor in lipid metabolism and may explain the lack of the statistical differences within exercise groups (RT+MICT and RT+HIIT) on TG and HDL-C. Previous studies with a similar exercise mode and intensity design but with higher intervention length (from 4 to 8 months) showed significant effects on TC, LDL-C, HDL-C and TG (Choi et al., 2015; Currie et al., 2015; Theodorou et al., 2016). Similarly, our training program did not induce any significative change in SBP, DBP, MBP, and hs-CRP across groups. Previous studies with hypertensive older adults (Guirado et al., 2012; Dos Santos et al., 2014; Theodorou et al., 2016) using a similar exercise design but with higher intervention lengths (4, 6, and 8 months, respectively), positively improved SBP, DBP and hs-CRP. Therefore, the short-term length in our trial may have limited and confounded some of the blood pressure outcomes.

Lastly, the intervention groups significantly decreased WC without any significant change in other morphologic outcomes (BW, FM, and FFM). Nevertheless, the decrease in visceral adiposity is an important health risk factor due to its association with the increase in insulin resistance- a major marker of the unbalance of glucose metabolism, CVD and T2D (De Koning et al., 2007; Canoy, 2008). Notably, both concurrent training programs (RT+HIIT or RT+MICT) were ineffective to increase FFM, possibly due to the low RT intensity and RT volume. After 12-weeks of exercise training, Robinson et al. (2017) showed significant improvements in FFM using higher volumes (MICT, 30 min at 70% VO2peak, 5 days/week; RT, 2–4 sets per exercise, 4 times/week). Similarly, 12-weeks of RT+HIIT program (3 times/week; HIIT, 26–30 min, 50 m running interleaved by 350–150 ms of rest; RT, 2 sets of 10 exercises at 20:40–40:20 work ratio; and with maximal intensity) increased FFM and muscle mass, and decreased BM, FM and BMI (García-Pinillos et al., 2019). Emerging evidence highlight that RT (Morton et al., 2019) and HIIT (Bishop et al., 2019) volume are key factors to evoke muscle mass response.

The present study has several strengths including the randomized design, the use of well-validated instruments and the wide range of MetS outcomes objectively measured- an important methodological issue in a high-risk population. In contrast, the short-term design, the scarce nutritional and pharmacological control (Supplementary Table S1) and the relative heterogeneous age range sample may have confounded some of the results, particularly on lipid and blood pressure outcomes. Therefore, the causality relationship should be carefully interpreted due to the small sample size within each group and to the wide age range. We tried to mitigate these limitations adopting specific statistical procedures to counteract these effects, controlling for several covariates that were available and evaluated as potential confounders. In addition, we calculated the magnitude of the effects size with the Cohen *d* effect size to accurately interpret our results. Unfortunately, residual confounding factors due to unknown or incompletely measured factors cannot be excluded. Thus, future studies on this topic should use a long-term design and control for nutritional and pharmacological treatment. Despite these limitations, our results have important clinical implications once we showed that both forms of concurrent training counteract the deleterious effects of MetS in older adults. Further, our results suggest that RT+HIIT may promote higher gains and present more pronounced effects to manage several important metabolic outcomes than RT+MICT, although these results need empirical confirmation of long-term exercise interventions.

## Conclusion

The findings of the present study suggest that both modes of concurrent training (i.e. RT+HIIT and RT+MICT) provide multiple cardiometabolic benefits in adults and older adults with MetS after a 12-week intervention. Further, concurrent training combining both aerobic and RT at high intensity interval training can potentially provide higher gains, enhance FBG and insulin sensitivity in people with MetS. However, more empirical evidence from long-term intervention studies is needed to confirm these results. Moreover our results suggest that both intensity and mode of intervention are fundamental factors to consider when designing and prescribing an exercise programs in adults and older adults with MetS. Therefore, these results may provide further support and may guide fitness professionals and exercise physiologists on exercise prescription process in this high-risk population.

## Data Availability Statement

The datasets generated for this study are available on request to the corresponding author.

## Ethics Statement

All methods and procedures were approved by the Ethic Committee of the Faculty of Sport Sciences and Physical Education of the University of Coimbra (FCDEF, reference: CE/FCDEF-UC/00202016). The patients/participants provided their written informed consent to participate in this study.

## Author Contributions

MD, HL, MV, and RM designed the study, analyzed the data, and wrote the manuscript. LB, RN, ED, FL, and EC added important intellectual content by criticizing and correcting previous versions of the manuscript. All authors approved the final version of the manuscript.

## Conflict of Interest

The authors declare that the research was conducted in the absence of any commercial or financial relationships that could be construed as a potential conflict of interest.
