# Supplementary material for: The Effects of Concurrent Training Combining Both Resistance Exercise and High-Intensity Interval Training or Moderate-Intensity Continuous Training on Metabolic Syndrome
Source: Front Physiol. 2020 Jun 11;11:572. doi: 10.3389/fphys.2020.00572 (PMC7300209; doi:10.3389/fphys.2020.00572)
Supplement: Supplementary file 1 [file Data_Sheet_1.docx]

Supplemental figures from changes in the participants' clinical characteristics after 12 weeks of resistance training plus high intensity interval training (RT+HIIT) or resistance training plus moderate intense continuous training (RT+MICT). The figures were created using the GraphPad Prism v6.


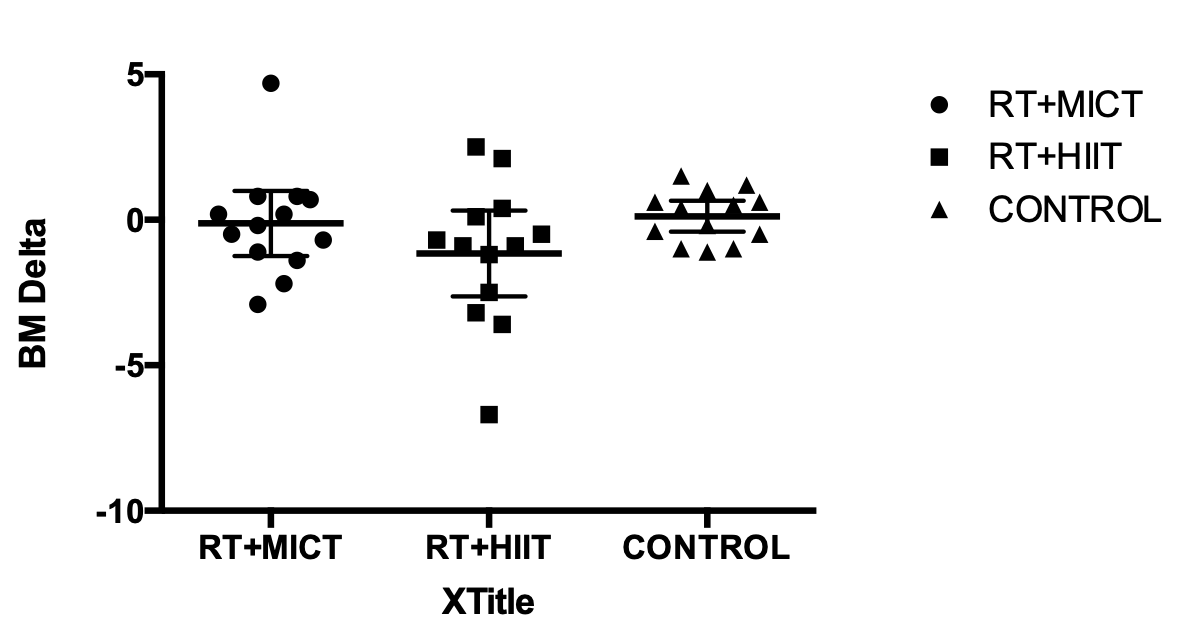


Figure S1: Delta change in body mass (BM; Kg) after 12 weeks of resistance training plus high intensity interval training (RT+HIIT) or resistance training plus moderate intense continuous training (RT+MICT). The horizontal lines represent mean and 95% confidence intervals, while each symbol (ball, square and triangle) represents a participant.


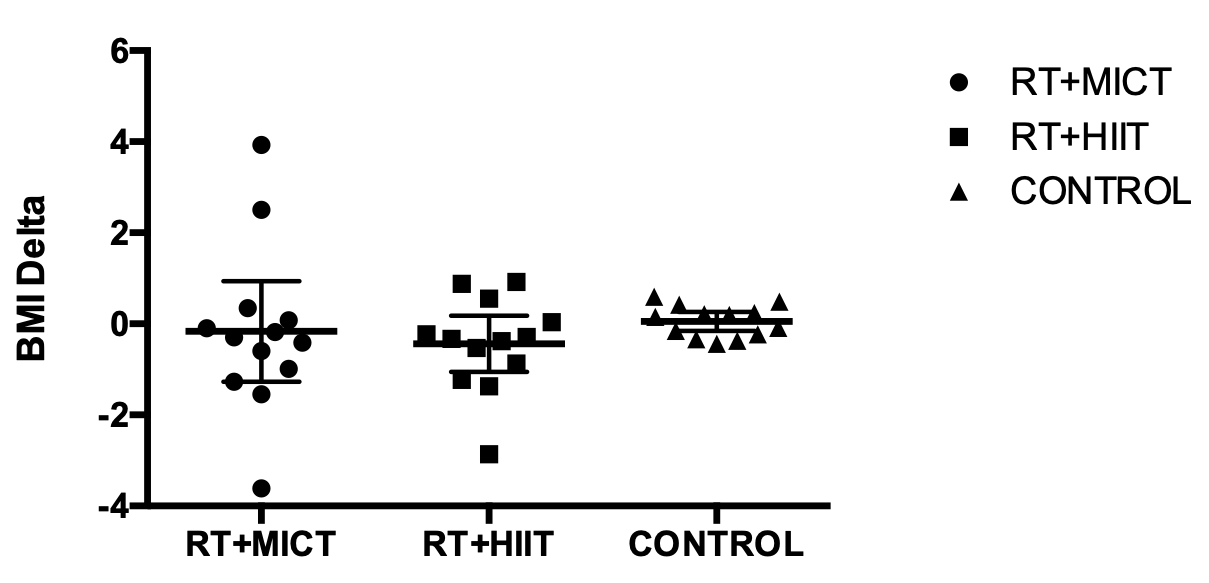


Figure S2: Delta change in body mass index (BMI; Kg/m2) after 12 weeks of resistance training plus high intensity interval training (RT+HIIT) or resistance training plus moderate intense continuous training (RT+MICT). The horizontal lines represent mean and 95% confidence intervals, while each symbol (ball, square and triangle) represents a participant.


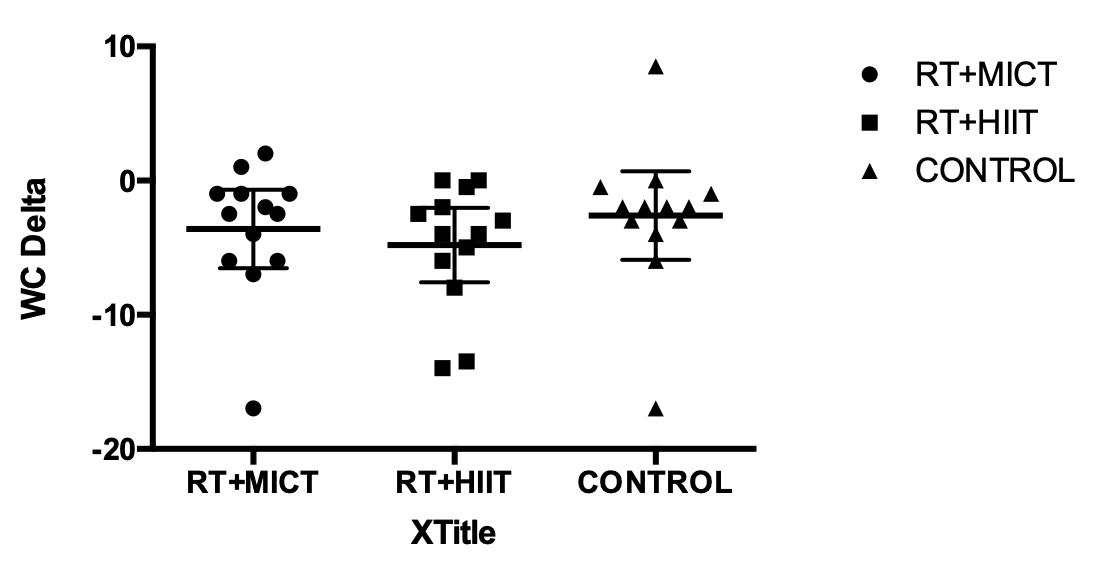


Figure S3: Delta change in waist circumference (WC; cm) after 12 weeks of resistance training plus high intensity interval training (RT+HIIT) or resistance training plus moderate intense continuous training (RT+MICT). The horizontal lines represent mean and 95% confidence intervals, while each symbol (ball, square and triangle) represents a participant.


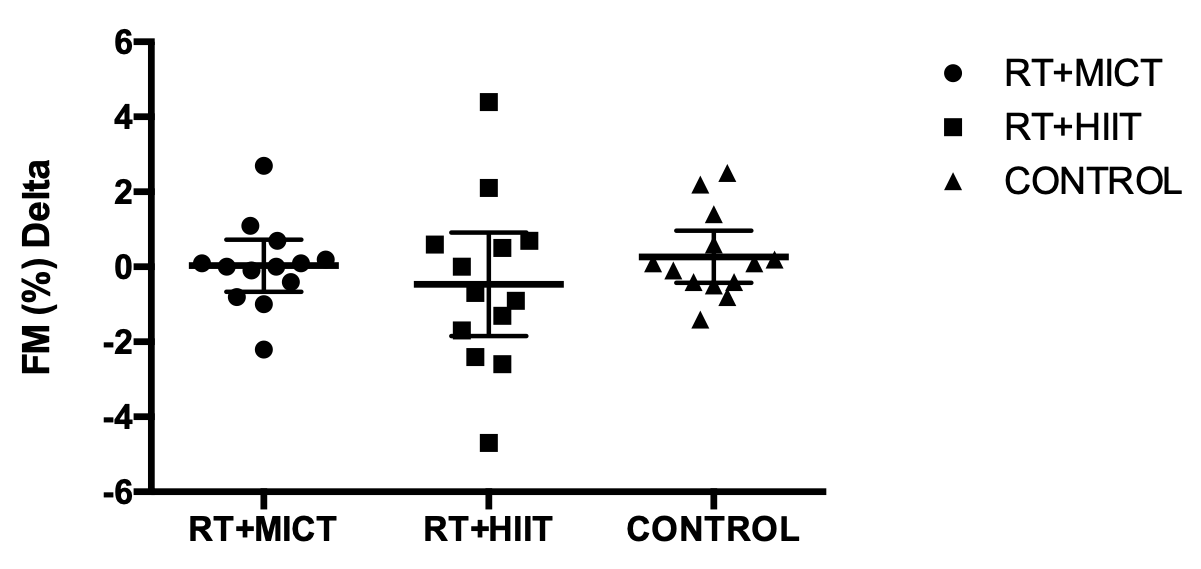


Figure S4: Delta change in fat mass (FM; %) after 12 weeks of resistance training plus high intensity interval training (RT+HIIT) or resistance training plus moderate intense continuous training (RT+MICT). The horizontal lines represent mean and 95% confidence intervals, while each symbol (ball, square and triangle) represents a participant.


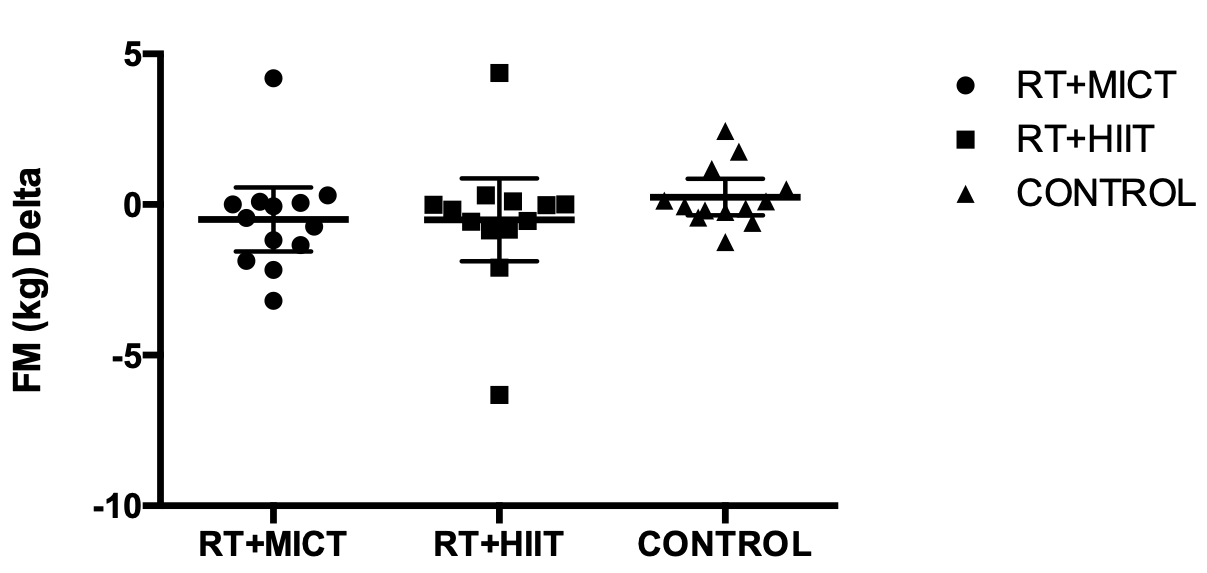


Figure S5: Delta change in fat mass (FM; Kg after 12 weeks of resistance training plus high intensity interval training (RT+HIIT) or resistance training plus moderate intense continuous training (RT+MICT). The horizontal lines represent mean and 95% confidence intervals, while each symbol (ball, square and triangle) represents a participant.


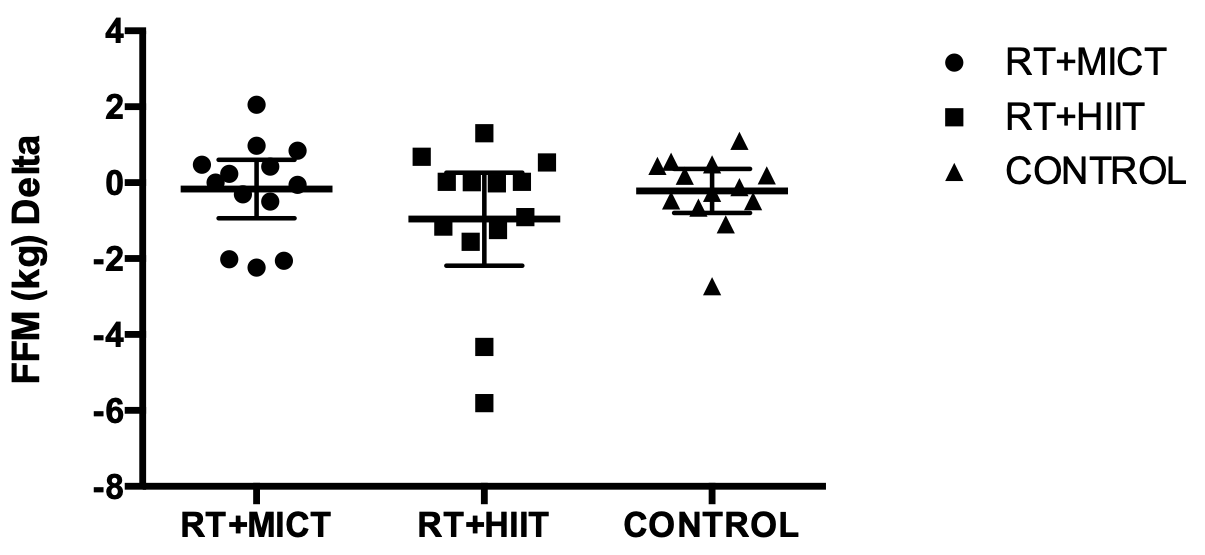


Figure S6: Delta change in fat free mass (FFM; Kg) after 12 weeks of resistance training plus high intensity interval training (RT+HIIT) or resistance training plus moderate intense continuous training (RT+MICT). The horizontal lines represent mean and 95% confidence intervals, while each symbol (ball, square and triangle) represents a participant.


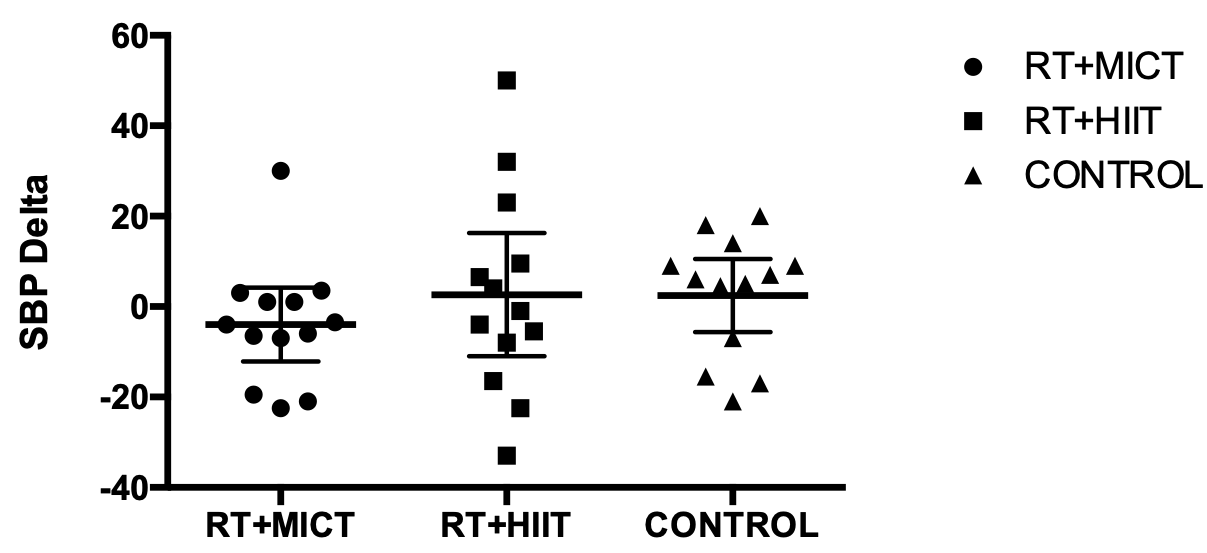


Figure S7: Delta change in systolic blood pressure (SBP; mmHg) after 12 weeks of resistance training plus high intensity interval training (RT+HIIT) or resistance training plus moderate intense continuous training (RT+MICT). The horizontal lines represent mean and 95% confidence intervals, while each symbol (ball, square and triangle) represents a participant.


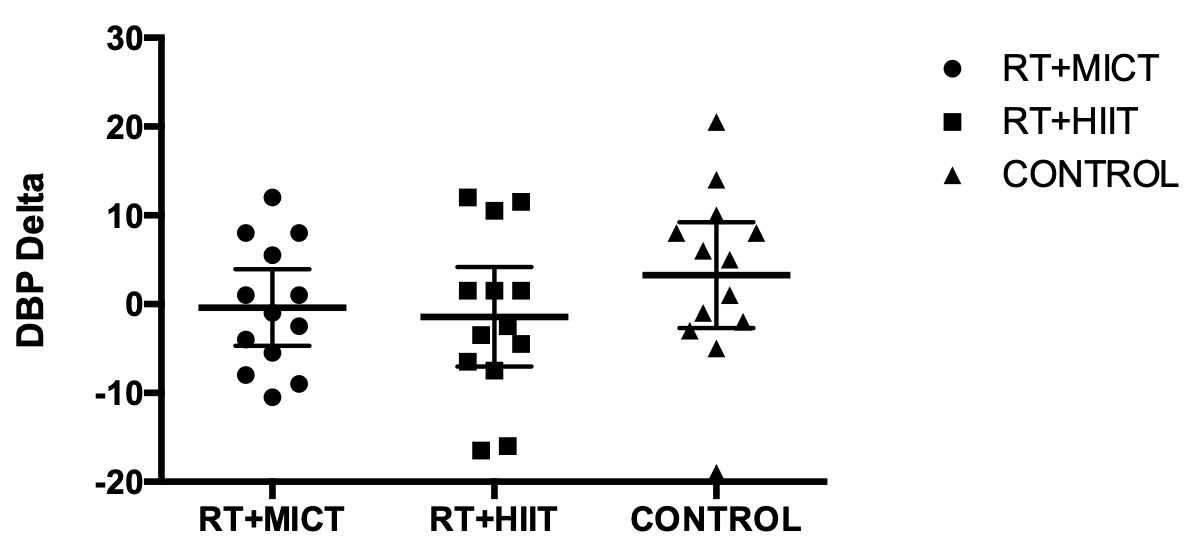


Figure S8: Delta change in diastolic blood pressure (DBP; mmHg) after 12 weeks of resistance training plus high intensity interval training (RT+HIIT) or resistance training plus moderate intense continuous training (RT+MICT). The horizontal lines represent mean and 95% confidence intervals, while each symbol (ball, square and triangle) represents a participant.


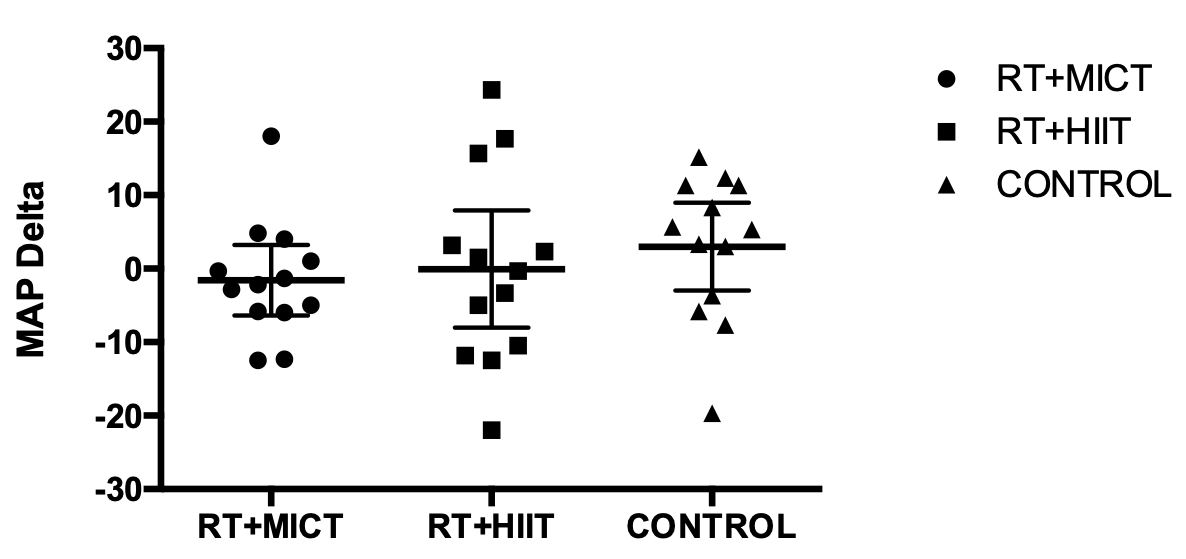


Figure S9: Delta change in Mean arterial pressure (MAP; mmHg) after 12 weeks of resistance training plus high intensity interval training (RT+HIIT) or resistance training plus moderate intense continuous training (RT+MICT). The horizontal lines represent mean and 95% confidence intervals, while each symbol (ball, square and triangle) represents a participant.


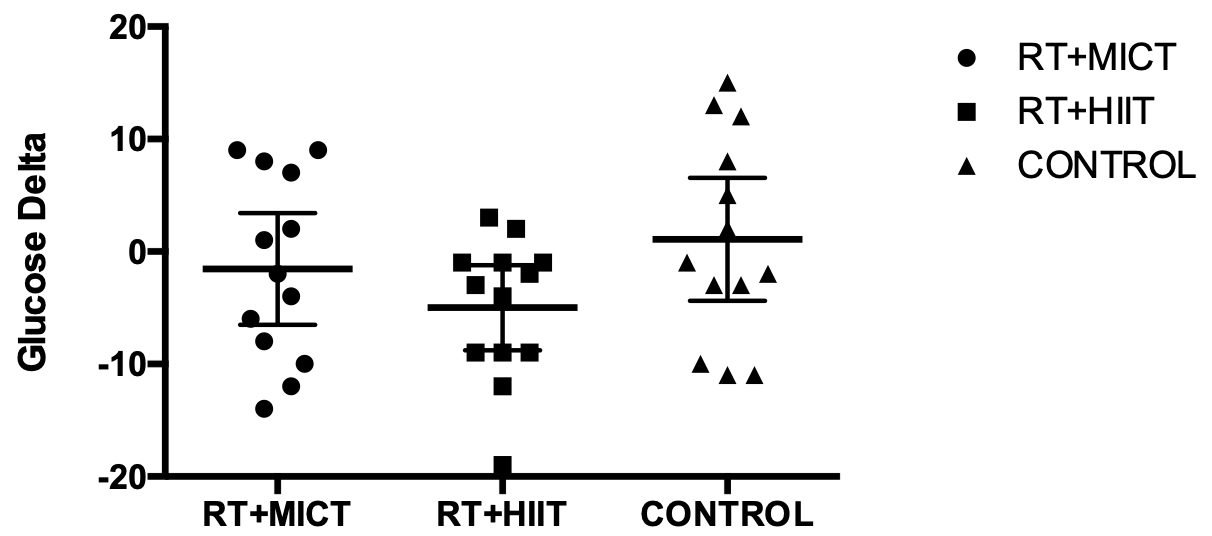


Figure S10: Delta change in fasting blood glucose (mg/dl) after 12 weeks of resistance training plus high intensity interval training (RT+HIIT) or resistance training plus moderate intense continuous training (RT+MICT). The horizontal lines represent mean and 95% confidence intervals, while each symbol (ball, square and triangle) represents a participant.


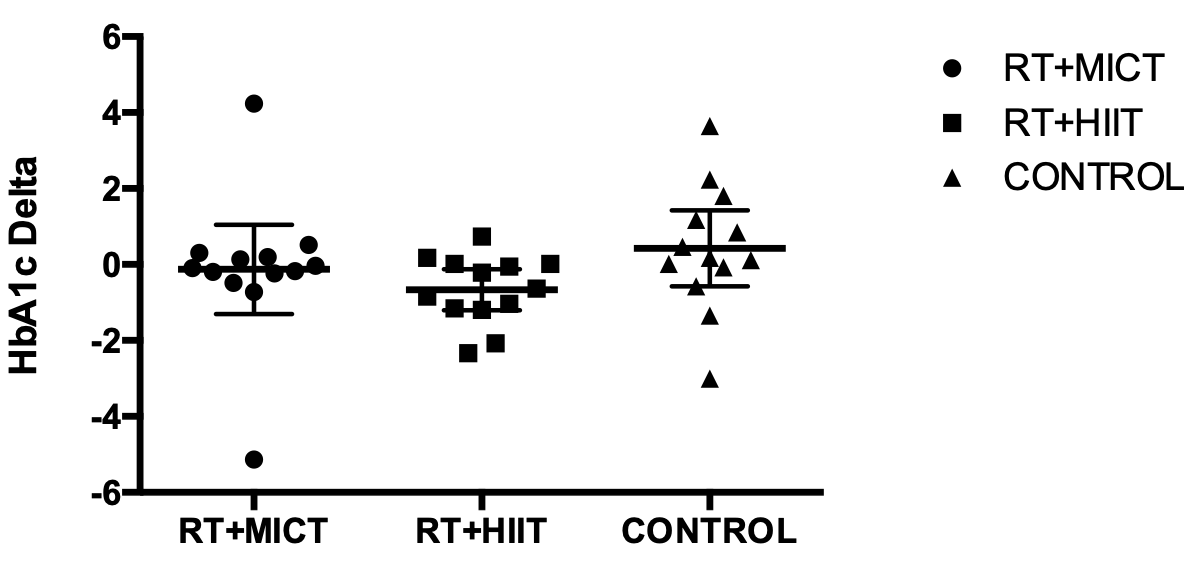


Figure S11: Delta change in glycosylated hemoglobin (HbA1c; %) after 12 weeks of resistance training plus high intensity interval training (RT+HIIT) or resistance training plus moderate intense continuous training (RT+MICT). The horizontal lines represent mean and 95% confidence intervals, while each symbol (ball, square and triangle) represents a participant.


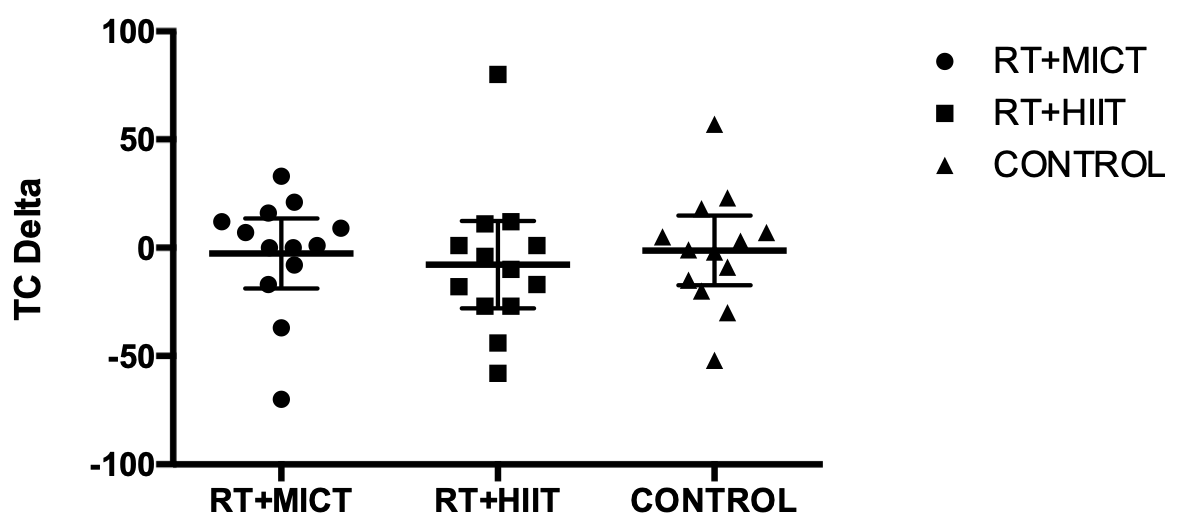


Figure S12: Delta change in total cholesterol (TC; mg/dl) after 12 weeks of resistance training plus high intensity interval training (RT+HIIT) or resistance training plus moderate intense continuous training (RT+MICT). The horizontal lines represent mean and 95% confidence intervals, while each symbol (ball, square and triangle) represents a participant.


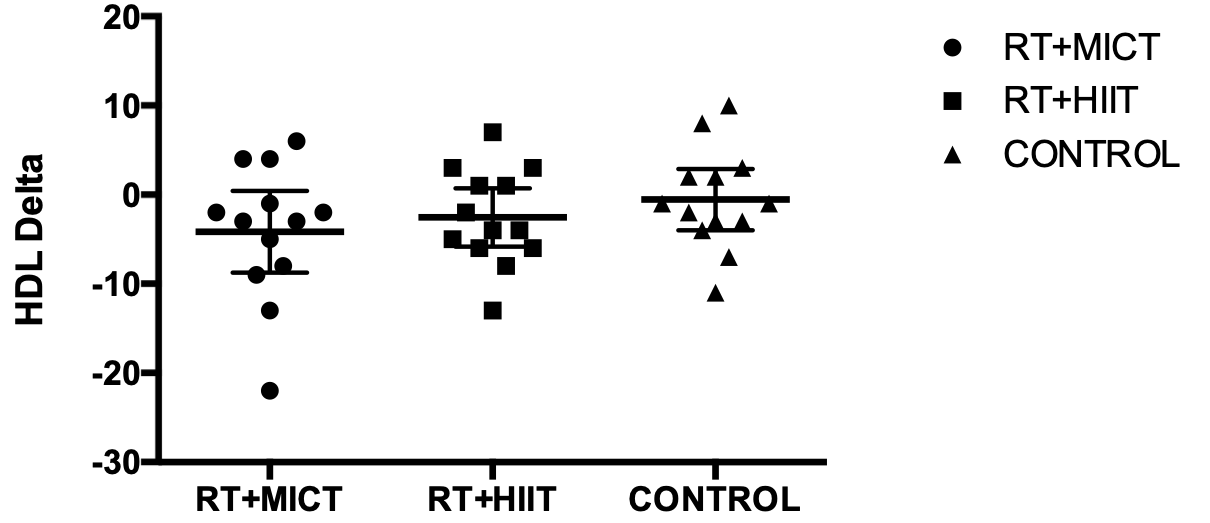


Figure S13: Delta change in high density lipoprotein (HDL; mg/dl) cholesterol after 12 weeks of resistance training plus high intensity interval training (RT+HIIT) or resistance training plus moderate intense continuous training (RT+MICT). The horizontal lines represent mean and 95% confidence intervals, while each symbol (ball, square and triangle) represents a participant.


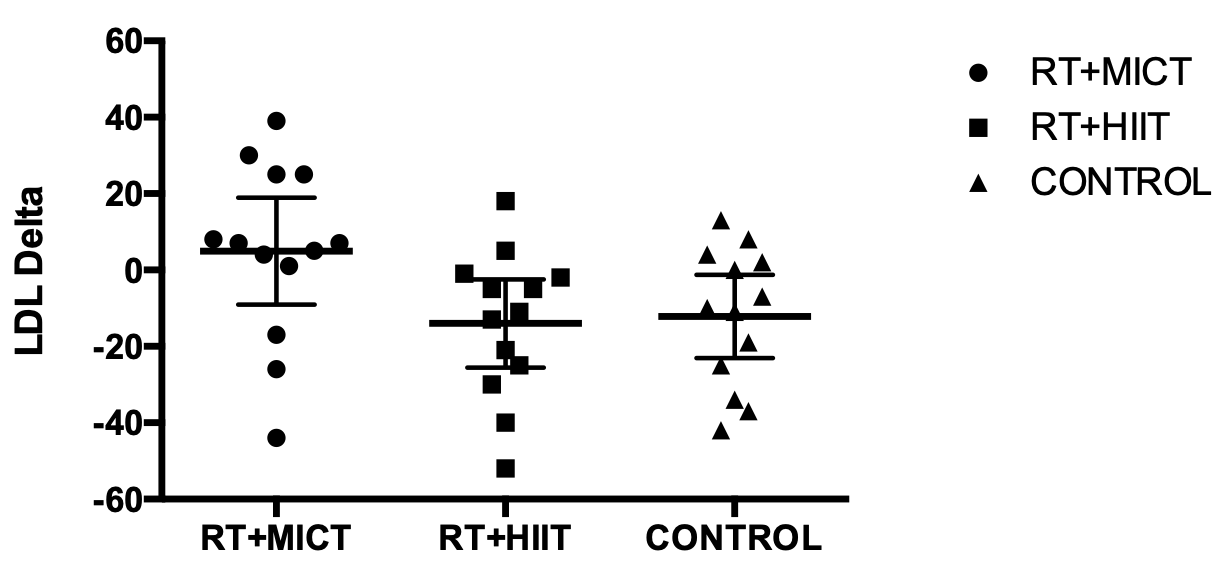


Figure S14: Delta change in low density lipoprotein (LDL; mg/dl) cholesterol after 12 weeks of resistance training plus high intensity interval training (RT+HIIT) or resistance training plus moderate intense continuous training (RT+MICT). The horizontal lines represent mean and 95% confidence intervals, while each symbol (ball, square and triangle) represents a participant.


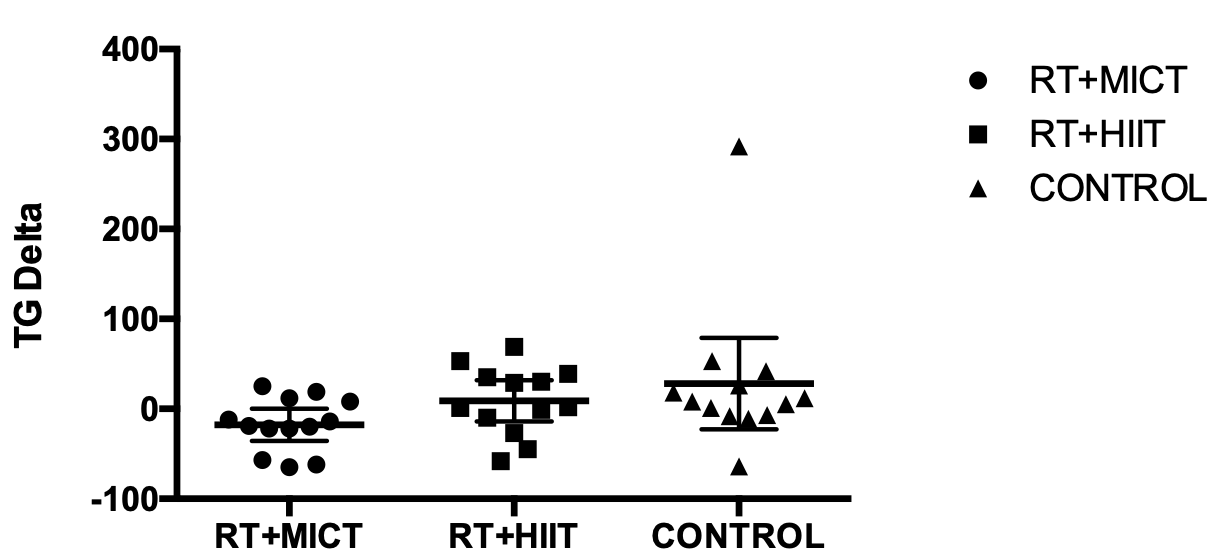


Figure S15: Delta change in triglycerides (TG; mg/dl) after 12 weeks of resistance training plus high intensity interval training (RT+HIIT) or resistance training plus moderate intense continuous training (RT+MICT). The horizontal lines represent mean and 95% confidence intervals, while each symbol (ball, square and triangle) represents a participant.


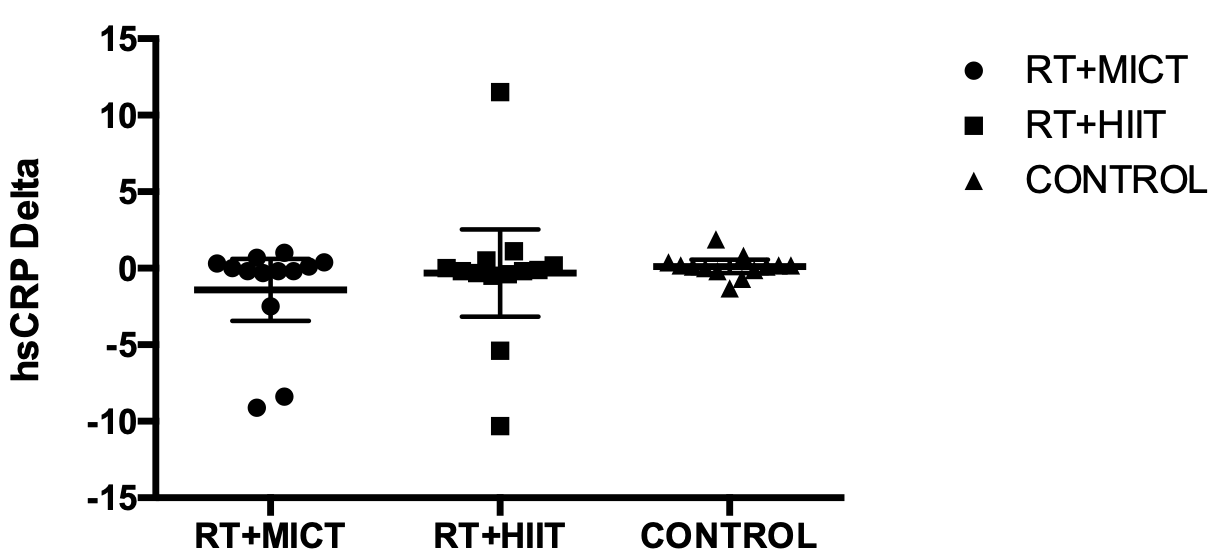


Figure S16: Delta change in high-sensitivity C-reactive protein (hsCRP; mg/dl) after 12 weeks of resistance training plus high intensity interval training (RT+HIIT) or resistance training plus moderate intense continuous training (RT+MICT). The horizontal lines represent mean and 95% confidence intervals, while each symbol (ball, square and triangle) represents a participant.


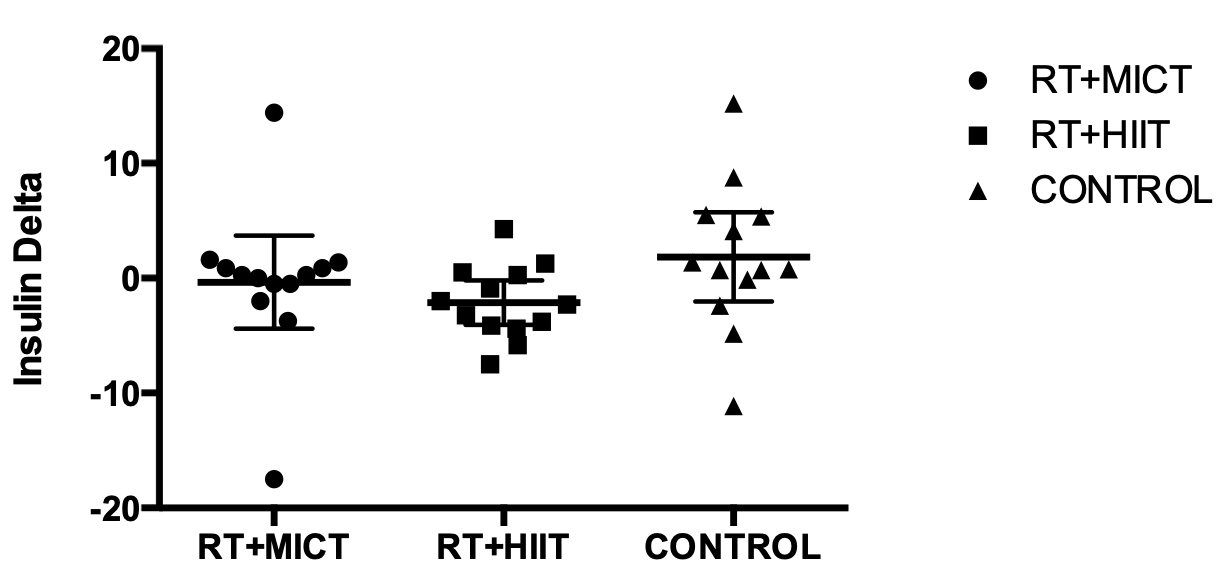


Figure S17: Delta change in insulin (mUI/l) after 12 weeks of resistance training plus high intensity interval training (RT+HIIT) or resistance training plus moderate intense continuous training (RT+MICT). The horizontal lines represent mean and 95% confidence intervals, while each symbol (ball, square and triangle) represents a participant.


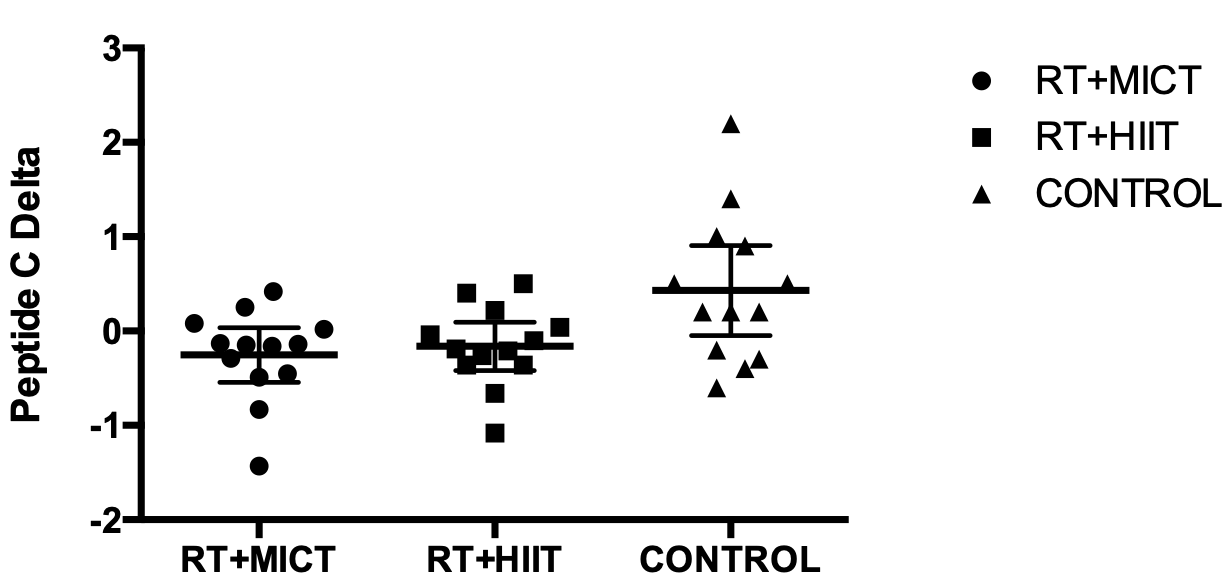


Figure S18: Delta change in Peptide C (ng/ml) after 12 weeks of resistance training plus high intensity interval training (RT+HIIT) or resistance training plus moderate intense continuous training (RT+MICT). The horizontal lines represent mean and 95% confidence intervals, while each symbol (ball, square and triangle) represents a participant.


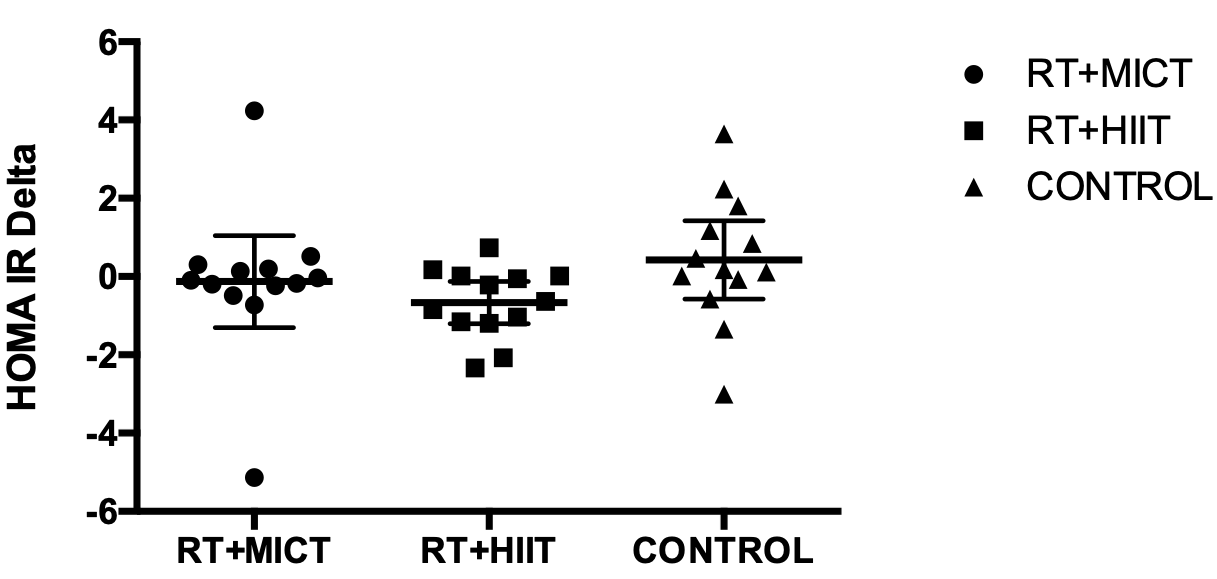


Figure S19: Delta change in HOMA-IR after 12 weeks of resistance training plus high intensity interval training (RT+HIIT) or resistance training plus moderate intense continuous training (RT+MICT). The horizontal lines represent mean and 95% confidence intervals, while each symbol (ball, square and triangle) represents a participant.
