# Supplementary material for: The Effects of Concurrent Training Combining Both Resistance Exercise and High-Intensity Interval Training or Moderate-Intensity Continuous Training on Metabolic Syndrome
Source: Front Physiol. 2020 Jun 11;11:572. doi: 10.3389/fphys.2020.00572 (PMC7300209; doi:10.3389/fphys.2020.00572)
Supplement: Supplementary file 2 [file Data_Sheet_2.docx]

Table S1: Medications.

| **Medication for disease (total)** | **RT+MICT (n = 13)** | **RT+HIIT (n = 13)** | **CON (n = 13)** | ***P*** |
| --- | --- | --- | --- | --- |
| Hypertension (24) | 9 | 9 | 6 | 0.377# |
| Diabetes (5) | 1 | **2** | 2 | 1.000£ |
| Dyslipidemia (15) | 7 | 7 | 1 | **0.020#** |
| Hypothyroidism (2) | 1 | 0 | 1 | 1.000£ |
| Beta-blockers (3) | 1 | 0 | 2 | 0.756£ |
| Anticoagulants (3) | 3 | 0 | 0 | 0.094£ |
| Angina pectoris (2) | 2 | 0 | 0 | 0.315£ |

# chi-square test; £, Monte Carlo test.
